# Characterization of the Structure and Immunostimulatory Activity of a Vaccine Adjuvant, De-*O*-Acylated Lipooligosaccharide

**DOI:** 10.1371/journal.pone.0085838

**Published:** 2014-01-22

**Authors:** Ji Eun Han, Seo Ri Wui, Kwang Sung Kim, Yang Je Cho, Wan Je Cho, Na Gyong Lee

**Affiliations:** 1 Department of Bioscience & Biotechnology, College of Bioscience, Sejong University, Seoul, Republic of Korea; 2 Research & Development Center, EyeGene, Seoul, Republic of Korea; 3 Yonsei University Gangnam Severance Hospital, Seoul, Republic of Korea; KAIST, Graduate School of Medical Science & Engineering, Republic of Korea

## Abstract

Lipopolysaccharide (LPS) is a major component of the outer membrane of Gram-negative bacteria. LPS elicits strong immunopathological responses during bacterial infection, and the lipid A moiety of LPS is responsible for this immunostimulatory activity. Lipid A exerts its biological activity by sending signals via TLR4 present on immune cells, and TLR4 agonists have been a target for vaccine adjuvant. Previously, we demonstrated an adjuvant activity of deacylated lipooligosaccharide (dLOS) to viral and bacterial antigens. In this study, we characterized the chemical structure of dLOS and evaluated its immunostimulatory activity on mouse and human immune cells in comparison with monophosphoryl lipid A (MPL). dLOS consists of a core oligosaccharide lacking the terminal glucose residue, a glucosamine disaccharide with two phosphate groups, and two *N*-linked acyl groups. dLOS was similar to MPL in induction of cytokine production in mouse peritoneal macrophages, but was a more potent activator in human monocytes and dendritic cells (DCs). Results of an analysis of allogeneic T cell responses revealed that dLOS induces Th1, Th2, and Th17-type immune responses in a dose-dependent manner. The immunostimulatory activities of dLOS were completely abrogated in *TLR4^−/−^* mice, which confirms its TLR4-dependency. These results suggest that in the presence of the core oligosaccharide, *O*-linked acyl groups of LPS are dispensable for activating the TLR4 signaling pathway. dLOS did not cause any pathological effects or death at 0.25, 0.5, or 1 mg per kg body weight in mice in the acute toxicity tests. This result suggests that dLOS has a low toxicity. dLOS should be considered for further development as a safe and effective adjuvant for human vaccines.

## Introduction

Lipopolysaccharide (LPS) is a major component of the outer membrane of Gram-negative bacteria. It is a potent stimulator of various immune cells and triggers the innate immune response. LPS is composed of three domains, an amphipathic domain known as lipid A, a core oligosaccharide (OS), and an O-antigenic polysaccharide. Lipid A is responsible for the endotoxic activity of LPS and exerts an immune stimulatory effect via toll-like receptor 4 (TLR4) signaling of diverse types of immune cells [1]. It activates antigen presenting cells by inducing cytokine secretion, co-stimulatory molecule expression, and antigen presentation, which links innate immune response to adaptive response [2], [3]. Lipid A derivatives with reduced toxicity have been targets for the development of human vaccine adjuvant. Monophosphoryl lipid A (MPL) is a non-toxic derivative of LPS isolated from *Salmonella minnesota* rough strain. MPL, in combination with aluminum salt, has been approved for use as an adjuvant for hepatitis B virus (HBV) and human papillomavirus (HPV) vaccines [4], [5]. Several other synthetic structural analogs of lipid A have been prepared to obtain TLR4 agonists with reduced toxicity [6], [7].

LPS-derivatives, including lipid A-like molecules, greatly vary in their biological activity. Their functions are influenced by lipid A structural variation, the number of phosphate groups on lipid A, and the symmetry, number, and length of the fatty acyl chains [8], [9]. The core OS moiety of LPS also affects the biological activity [10], [11]. Previously, we prepared lipooligosaccharide (LOS) from an *Escherichia coli* rough strain that expresses LPS lacking O-antigen, and obtained de-acylated lipooligosaccharide (dLOS) by alkaline hydrolysis [12]. dLOS was evaluated for adjuvant activity to several vaccine antigens. It markedly increased antibody responses to HBV surface antigen (HBsAg), but also enhanced interferon (IFN)-γ production by mouse splenocytes. This result indicated that dLOS promotes a Th1-type cellular immune response as well as a Th2-type antibody response [13]. Combining dLOS and aluminum hydroxide (alum) synergizes their adjuvant effects to HPV L1 VLPs and anthrax protective antigen (PA), which suggests that this combination has potential as a good vaccine adjuvant [14]–[17]. In this study, we determined the chemical structure of dLOS and investigated the immunostimulatory activity of dLOS compared to MPL in mouse and human immune cells. We also evaluated the toxicity and pyrogenicity of dLOS in mice and rabbits, respectively.

## Materials and Methods

### Ethics

Animal experiments were reviewed and approved by the Institutional Review Committees of Sejong University. Collection of human blood from healthy donors were reviewed and approved by the Institutional Review Committees of Gangnam Severance Hospital of Yonsei University, and written informed consent was obtained from all the participants.

### Mice and reagents

Six-week-old specific pathogen-free female BALB/c or C57BL/6 mice were purchased from Japan SLC (Hamamatsu, Japan) or DBL (Chungcheongbuk-do, Korea). *TLR4^−/−^* BALB/c mice were kindly provided by Dr. M. Kwon (International Vaccine Institute, Seoul, Korea) with permission from Prof. S. Akira (Osaka University, Osaka, Japan). LPS from *E. coli* O111:B4 and MPL from *S. minnesota* R 595 were purchased from Sigma-Aldrich (St. Louis, MO, USA). Kdo_2_-lipidA, synthetic glucopyranosyl lipid adjuvant (GLA), and detoxified lipid A from *S. minnesota* R595 were obtained from Avanti Polar Lipids (Alabaster, AL, USA). Aluminum hydroxide (Alhydrogel®) was obtained from Brenntag Biosector (Frederikssund, Denmark). Endotoxin activity was determined using the Endosafe®-Portable Test System (PTS) (Charles River Laboratories, Wilmington, MA, USA).

Human recombinant granulocyte-macrophage colony-stimulating factor (GM-CSF) and interleukin (IL)-4, and mouse recombinant IL-2, IL-4, and GM-CSF were purchased from R&D systems (Minneapolis, MN, USA). Cytokine ELISA kits were from R&D Systems or BD Biosciences (San Jose, CA, USA). Mouse anti-human CD14 monoclonal antibody (mAb)-fluorescein isothiocyanate (FITC), anti-CD80 mAb-FITC, anti-CD86 mAb- phycoerythrin (PE), and anti-HLA-DR mAb-PE were purchased from BD Biosciences. Anti-mouse CD11c mAb-FITC, anti-CD40 mAb-PE, anti-CD80 mAb-PE, and anti-CD86 mAb-PE were also obtained from BD Biosciences. Mouse anti-LPS core mAb (clone WN1 222-5) was purchased from Avanti Polar Lipids. Bovine serum albumin (BSA) was purchased from Santa Cruz (Dallas, Texas, USA). Cell culture media and antibiotics were obtained from WelGene (Daegu, Korea), and fetal bovine serum (FBS) was from Gibco/Invitrogen (Carlsbad, CA, USA).

### Preparation of de-*O*-acylated lipooligosaccharide

LOS was isolated from an *E. coli* strain that expresses LPS lacking O-polysaccharide. Purification and deacylation of LOS was performed as previously described, with minor modifications [12], [18]. Briefly, bacterial cells were treated three times with acetone, and LOS was purified by phenol/chloroform/petroleum ether extraction. Deacylation was performed by treating the LOS with 0.1 N NaOH at 60°C for 2 h. The pH of the reaction mixture was adjusted to 7.0 by the addition of 1 N acetic acid, and dLOS was precipitated with cold anhydrous ethanol. The dLOS pellet was washed three times with ethanol, and the released fatty acid was removed by dialysis. dLOS was quantified using the 2-keto-3-deoxyoctonate assay as described by Lee *et al.* [19], and the reduced size of the dLOS was visualized on a silver-stained SDS-polyacrylamide gel.

### MALDI-TOF analysis

Samples were dissolved in distilled water (5 mg/ml), and were then mixed with matrix (2,5-dihydroxybenzoid acid; Sigma). MALDI-TOF analysis was performed using an Axima-LNR V2.1.0 mass spectrometer (Shimadzu, Kyoto, Japan) at the Carbohydrate Research Center, Sejong University.

### Immunoblot analysis

Samples were resolved on SDS-gradient polyacrylamide gels (EzWay PAG Pre-cast gel, 4–12%, Koma Biotech, Seoul, Korea) using an X-cell II™ Mini-Cell (Invitrogen, Carlsbad, CA, USA), and blotted onto polyvinylidene difluoride (PVDF) membranes (Invitrogen) in transfer buffer (25 mM Tris⋅Cl, pH 8.3, 192 mM glycine, 20% v/v methanol) for 1 h at 100 V. Membranes were blocked in 5% skim milk in phosphate-buffered saline (PBS, pH 7.3) containing 0.05% tween-20 (PBST), overnight, and probed with anti-LPS core mAb for 2 h at room temperature. Bound antibodies were detected by incubating with goat anti-mouse IgG antibody conjugated with horseradish peroxidase (ThermoFisher Scientific, Waltham, MA, USA), followed by development with the peroxidase substrate DAB kit (Vector Laboratories, Burlingame, CA, USA). LPS derivatives were also confirmed by silver-staining of SDS-polyacrylamide gels.

### ELISA

Anti-LPS core mAb reactivity to LPS, dLOS, and lipid A derivatives was determined by ELISA. A 96-well immunoplate (ThermoFisher Scientific) was coated by overnight incubation at 4°C with 100 µl of samples that were serially diluted in PBS. Wells were washed four times with PBST and blocked with 300 µl of 1% BSA in PBS for 2 h at room temperature. The plate was incubated with anti-LPS core mAb for 2 h at room temperature. Bound antibodies were detected using horseradish peroxidase-conjugated goat anti-mouse IgG, followed by incubation with *o*-phenylenediamine dihydrochloride solution (SIGMA*FAST*™ OPD, Sigma-Aldrich) as a chromogenic substrate. A volume of 1 N H_2_SO_4_ (100 µl) was added to terminate the reaction, and absorbance at 492 nm was measured using an EL800 Universal microplate reader (Bio-Tek, Winooski, VT, USA).

### Measurement of cytokine production by peritoneal macrophages

Each BALB/c mouse received an intraperitoneal injection of 3% thioglycollate medium (2 ml). Three days after the injection, the peritoneal cavity was flushed with 10 ml ice-cold culture medium to collect macrophages. Cells were plated in 100-mm culture dishes and incubated for 1 h. Nonadherent cells were washed out two times with ice-cold PBS. Adherent cells were cultured at a density of 2.5×10^5^ cells/well in 96-well plates and were stimulated with LPS, MPL, dLOS, or medium alone for 24 h. Secreted cytokines were assessed using sandwich ELISA.

### Isolation and culture of human PBMC and monocytes

Human blood from healthy donors was collected in heparinized tubes. Peripheral blood mononuclear cells (PBMCs) were isolated on a Ficoll-Paque™ density gradient (GE HealthCare, Uppsala, Sweden). PBMCs were cultured at a density of 5×10^6^ cells/ml in 24-well plates in complete medium (RPMI1640 supplemented with L-glutamine, 10% FBS, and 25 mM HEPES). Monocytes were isolated from the PBMC fraction by positive selection using CD14 microbeads and a magnetic separator (MACS; Miltenyi Biotec, Bergisch Gladbach, Germany). Isolated CD14-positive monocyte purity was >85% (FACSCalibur flow cytometer, Becton Dickinson, Franklin Lakes, NJ, USA). Human PBMCs and monocytes were stimulated with LPS, MPL, or dLOS for 12 h and 24 h. Cell-free culture supernatants were collected, and the released cytokines were determined using a Duoset assay or OptEIA™ Set according to the manufacturer's protocol.

### Determination of mouse BMDC activation

Bone marrow (BM) cells were isolated from the hind leg long bones of BALB/c mice. After red blood cell lysis, cells were suspended at a density of 1×10^6^ cells/ml in RPMI1640 supplemented with 10% FBS, glutamine (2 mM), β-mercaptoethanol (5 µM), GM-CSF (10 ng/ml), and IL-4 (1 ng/ml). Cells were cultured for 7 days before they were harvested. Bone marrow-derived dendritic cells (BMDCs) were isolated by positive selection using CD11c microbeads and stimulated for 24 h with LPS, MPL, dLOS, or medium only. Cells were then stained for cell surface markers using the following antibodies: CD11c-FITC, CD40-PE, CD80-PE, and CD86-PE, followed by flow cytometry (FACSCalibur™ flow cytometer, Becton Dickinson). Acquisition and analysis of samples were performed using CELLQuestPro software (Becton Dickinson). CD11c-positive cells were gated, and expression of CD40, CD80, or CD86 was analyzed. The culture media with activated BMDCs were also assayed for cytokine levels using sandwich ELISA.

### Determination of human MDDC maturation

To obtain monocyte-derived dendritic cells (MDDCs), CD14-positive human monocytes were seeded at a density of 1×10^6^ cells/ml in 24-well plates and cultured in an IL-4 (1000 U/ml), GM-CSF (500 U/ml), and 1% nonessential amino acid supplement medium. One-half of the medium was replaced every 2 days. At day 6, immature dendritic cells (DCs) were induced into maturation by incubating with LPS, MPL, or dLOS for 24 h. Cells were then stained for cell surface marker expression using HLA-DR-PE, CD80-FITC, or CD86-PE antibodies. MDDCs were double-stained with anti-CD14-FITC mAb, followed by flow cytometry. CD14-negative cells were gated, and expression of HLA-DR, CD80, or CD86 was analyzed.

### Allogeneic T cell response assays

BMDCs prepared from C57BL/6 mice were stimulated with MPL or dLOS for 24 h. After washing with fresh medium, cells were seeded into 96-well cell culture plates. Naïve CD4-positive T cells were purified from the spleens of BALB/c mice using CD4^+^ T cell isolation kit II (Miltenyi Biotec). Stimulated BMDCs were added to the wells that contained CD4-positive T cells (2×10^5^ cells) at a ratio of 1∶10 or 1∶20 (BMDCs∶T cells), and were cultured for 5 days. Secretions of IFN-γ, IL-5, and IL-17 in culture supernatants were determined using sandwich ELISA.

### Assays for mouse serum cytokine levels

BALB/c mice (*n* = 3) were given an intramuscular injection with LPS (1 µg), MPL (1 or 5 µg), or dLOS (1 or 5 µg) alone, or in combination with alum at a 1∶50 ratio. Control mice were injected with saline. Blood was collected by cardiac puncture at 1 h or 4 h post-injection, allowed to clot at room temperature for 2 h, and then centrifuged. Individual serum samples were assessed for cytokine and chemokine levels using a multiplex cytokine assay (Milliplex MAP assay kit, Millipore, Billerica, MA, USA).

### Acute toxicity test

ChemOn Inc. (Suwon, Korea) followed good laboratory practice (GLP) guidelines to test the acute toxicity of dLOS using specific pathogen-free male and female ICR mice. Eight week-old mice (*n* = 5) were given an intramuscular injection with PBS or dLOS (0.25, 0.5, or 1.0 mg/kg body weight). Each mouse was monitored for changes in body weight and mortality. At day 14, each mouse was anesthetized using ether, and was then euthanized and a necropsy was performed.

### Pyrogenicity test

The pyrogenicity test of dLOS combined with alum was performed by Harlan Laboratories (Indianapolis, Indiana, USA) in accordance with European Pharmacopoeia 7^th^ edition guidelines under GLP conditions. The test was carried out using male New Zealand white rabbits (>3 kg). Each rabbit was weighed and secured in a rabbit restraint stock. The internal body temperature of each rabbit was recorded at 30 min intervals for at least 90 min before injection with the test material. Each rabbit was given an intravenous injection via the marginal ear vein with dLOS plus alum at 1/60 of a human dose in a volume of 1 ml per kg body weight. The temperature of each rabbit was then recorded at 30 min intervals for 3 h post-injection. The difference between the maximum post-dose temperature and the mean initial temperature was calculated.

### Statistical analysis

*P* values less than 0.05 were considered statistically significant. A two-tailed Student's *t*-test was used to compare two experimental groups.

## Results

### Characterization of dLOS chemical structure

**Figure 1:**
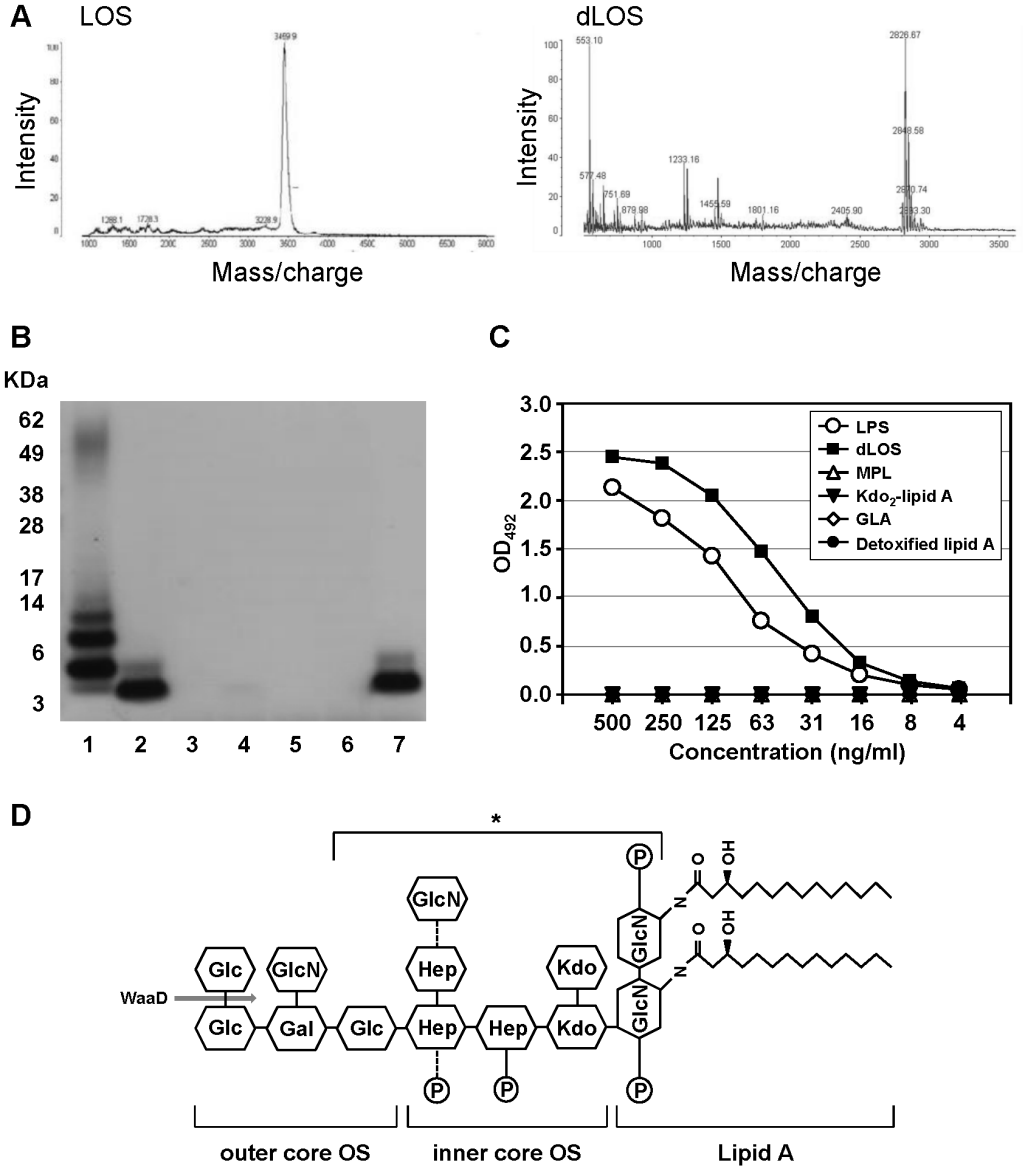
Characterization of dLOS structure. (A) Mass spectra for *E. coli* LOS and dLOS. (B) and (C) Reactivity of LPS, dLOS, and four lipid A derivatives with an mAb specific for the core OS region of LPS as determined by immunoblot analysis and ELISA, respectively. Lanes on the immunoblot: 1, LPS 2.5 µg; 2, dLOS 1 µg; 3, MPL 4 µg; 4, Kdo_2_-lipid A 4 µg; 5, GLA 4 µg; 6, detoxified lipid A from *S. minnesota* R595 4 µg; 7, dLOS 1 µg. The positions of protein size markers are marked on the left side of the gel. Data in B and C represent two and four independent experiments, respectively, with similar results. (D) The predicted chemical structure of dLOS. In the inner core OS, either glucosamine or phosphate is linked to the third heptose or the second heptose (marked with dotted lines), respectively. The gray arrow shows the site where the transfer of the terminal glucose on the outer core is blocked by the *waaD* gene mutation. The WN1 222-5 antibody binding site is denoted by an asterisk.

We performed a MALDI-TOF analysis to determine the molecular mass of dLOS. LOS purified from an *E. coli* rough strain yielded one major peak at 3459.9 *m/z* on a mass spectrum (Figure 1A). In contrast, the mass spectrum profile of dLOS exhibited several peaks: a predominant one at 2826.67 *m/z* and several minor ones at 2405.90 *m/z*, 1801.16 *m/z*, 1455.59 *m/z*, and 1233.16 *m/z*. Around the peak at 2826.67 *m/z*, multiple peaks appeared with 22 Da higher mass, indicating the presence of sodium adducts [M+Na]^+^. dLOS was prepared from LOS by alkaline hydrolysis, which is known to remove ester-linked fatty acids but leave amide-linked fatty acids intact [18]. The difference in molecular mass between LOS and dLOS was 633.23 Da, corresponding to three C-14 fatty acids. The lipid component of dLOS was determined using GC-mass analysis. After hydrolysis and esterification of fatty acids, derivatized acids were extracted in hexane, resolved in dry pyridine, and treated with BSTFA-TMCS. Analysis of the resulting product revealed that dLOS contains C-14, but not C-12, fatty acid (data not shown).

To investigate the LOS carbohydrate core length, we performed whole genome sequencing of the *E. coli* LPS mutant and the parent strain. Comparison of the genome sequences of the two strains revealed that the parent strain contained the *waaD* gene, which was completely deleted in the rough strain (data not shown). The *waaD* gene, encoding a UDP-glucose:(glucosyl) LPS α-1,2-glucosyltransferase, is present only in the *E. coli* R3 core-type strains, which suggested that dLOS contains the R3 core-oligosaccharide [20], [21]. A defect in this gene causes a loss of the terminal glucose on the outer core, which leads to an inability to ligate O-polysaccharide [20]. Therefore, mutant strain LOS is expected to contain only lipid A and core OS lacking the terminal glucose. We confirmed the presence of the core OS moiety by immunoblot analysis using mAb WN1 222-5, which is specific for the LPS core region [22]. WN1 222-5 bound to LPS and dLOS but not to four lipid A derivatives (Figure 1B). Gel results also clearly showed that dLOS is smaller in size than the lipid A-core OS of LPS. We used ELISA to compare the reactivity of WN1 222-5 with LPS, dLOS, and lipid A derivatives, and found that the antibody was highly reactive with LPS and dLOS but not with other lipid A derivatives (Figure 1C). A higher optical density of dLOS may be attributed to a greater molar ratio of dLOS compared with LPS. These results confirmed that dLOS retained the core OS moiety. Based on these data, we predicted the chemical structure of dLOS (Figure 1D). It contains the core OS devoid of the terminal glucose residue that includes a heptose-phosphate epitope and two Kdo residues, and the lipid A containing only two *N*-linked acyl groups and two phosphates. The molecular mass of dLOS calculated from the predicted structure was 2826.58 Da, which was in agreement with that obtained from MALDI-TOF analysis. The endotoxic activity of dLOS determined using the Endosafe®-Portable Test System was 5.4×10^3^ EU/mg, which was comparable to that of MPL.

### Comparison of the immunostimulatory activity of dLOS with MPL in mouse and human immune cells

**Figure 2:**
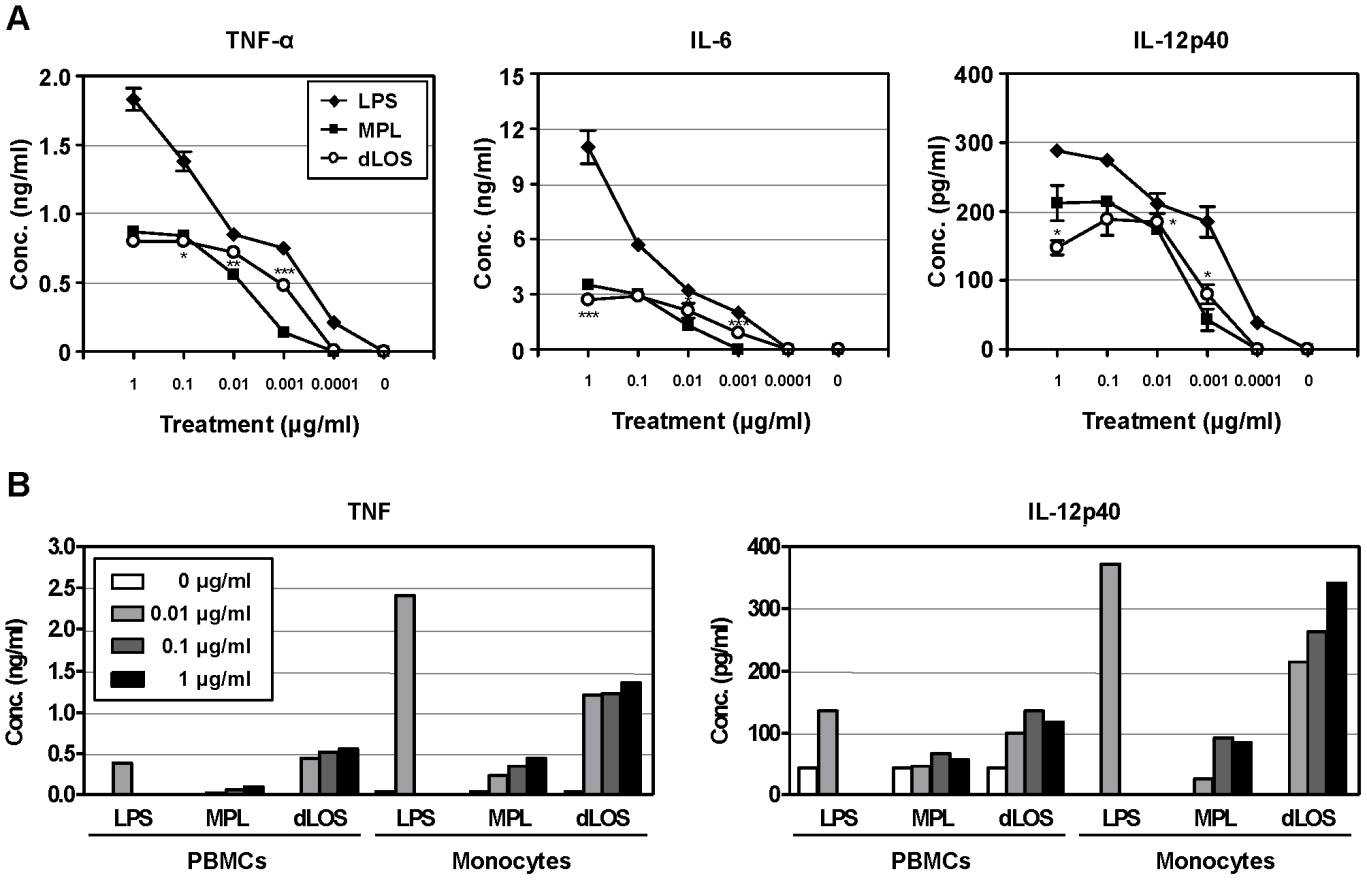
Responses of mouse macrophages and human monocytes to LPS, MPL, and dLOS. (A) Mouse peritoneal macrophages were cultured with LPS, MPL, or dLOS for 24 h. TNF-α, IL-6, and IL-12 levels released into culture media were determined by sandwich ELISA. Data are expressed as mean ± standard deviation (SD) of triplicate cultures and represent two independent experiments with similar results. ^*^, *P*<0.05; ^**^, *P*<0.01; ^***^, *P*<0.001 as compared with MPL-treated cells. (B) Human PBMCs and monocytes were cultured with LPS, MPL, or dLOS. Culture supernatant was harvested at 12 h or 24 h and assayed for TNF or IL-12 production, respectively. Data represent three independent experiments with similar results.

In our previous study, we assessed the ability of dLOS to induce cytokines in human whole blood cells, and showed that dLOS was less potent in stimulating tumor necrosis factor (TNF)-α release than LOS but retained the ability to trigger IL-12 production [12]. In this study to clarify dLOS immunostimulating activity, we compared LPS, dLOS, and MPL for activity on mouse and human primary immune cells. Mouse peritoneal macrophages were stimulated with LPS, dLOS, and MPL at various concentrations. Secreted levels of TNF-α, IL-6, and IL-12 were assessed (Figure 2A). In the LPS-stimulated macrophages, the levels of the three cytokines increased in a concentration-dependent manner up to 1 µg/ml and were significantly higher than those stimulated with dLOS or MPL. Immune responses to dLOS and MPL reached a plateau at 0.01 or 0.1 µg/ml. In general, responses to dLOS were slightly higher than MPL at low concentrations, but became lower at a concentration >0.1 µg/ml in terms of the ability to induce cytokine secretion.

The biological function of LPS, dLOS, and MPL were also examined in human immune cells (Figure 2B). When applied to PBMCs at 0.01 µg/ml, LPS and dLOS were similar in TNF and IL-12 induction and were higher than MPL. However, increased concentrations of dLOS did not increase the cellular response. Purified monocytes yielded different patterns in that LPS was more effective than dLOS in inducing both cytokines, and dLOS was more active than MPL. These results suggest that the immunostimulatory activity of dLOS is similar to MPL in mouse macrophages but is more active in human immune cells.

Because LPS is a strong stimulant of B cells, we also investigated whether dLOS retained the ability to activate B cells. Mouse splenocytes stained with carboxyfluorescein succinimidyl ester (CFSE) were stimulated with LPS, dLOS, or MPL. The intensity of CFSE expression on B220-postive cells was analyzed. The proliferation of B cells induced by dLOS was similar to LPS-treated cells, but was higher than MPL-treated cells (Figure S1). Increased expression of surface antigens on B220^+^ cells were also observed in these cells (Figure S2).

### DC maturation and activation by dLOS

**Figure 3:**
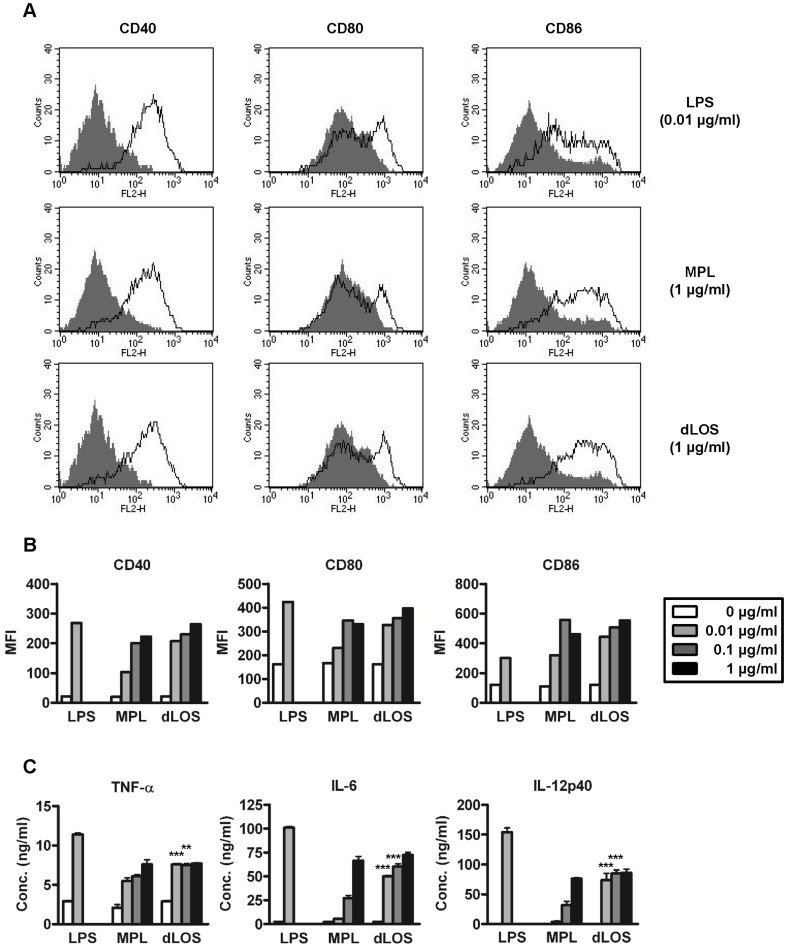
Increased expression of cell surface co-stimulatory molecules and cytokines in mouse BMDCs. (A) Mouse BMDCs were cultured for 24 h in the presence of LPS, MPL, or dLOS, and expression of CD40, CD80, or CD86 on CD11c-positive populations was assessed by flow cytometry (□). Unstimulated splenocytes are shown for comparison (▪). (B) Mean fluorescence intensity (MFI) for each molecule is shown as histograms. (C) Culture supernatants were assessed for TNF-α, IL-6, and IL-12 levels by sandwich ELISA. Values are the mean ± SD of triplicate cultures. ^**^ and ^***^, *P*<0.01 and *P*<0.001 as compared with MPL-treated cells, respectively. Data represent two independent experiments with similar results.

LPS acts as a potent inducer of DC activation and maturation via a TLR4 signaling pathway. This characteristic is important for the ability of adjuvants to link an innate immune response against vaccine antigens to an adaptive response. Previous studies reported that a TLR4 agonist retained biological activity when it consisted of at least 5 acyl groups on lipid A [9], [23]. Because dLOS has only two acyl groups, we investigated whether it can induce DC activation. Mouse BMDCs were stimulated with LPS, MPL, or dLOS. BMDC maturation status was determined by measuring the expression of the cell surface molecules, CD40, CD80, and CD86, on CD11c-positive cells. In a preliminary experiment, LPS induced a maximum response in surface molecule expression at 0.01 µg/ml. Therefore, we used 0.01 µg/ml LPS as a positive control and compared with dLOS and MPL at concentrations of 0.01–1.0 µg/ml. At 0.01 µg/ml, expression of all three surface molecules was higher in cells treated with dLOS than those treated with MPL. In both groups, however, the responses reached a plateau at 1.0 µg/ml, and no statistical difference was observed between the two groups (Figure 3A and 3B).

Cytokine secretion was also assessed to determine DC activation and maturation. We measured TNF-α, IL-6, and IL-12 levels in the supernatant of BMDCs stimulated with LPS, MPL, or dLOS (Figure 3C). All three cytokine responses were highest in the BMDCs treated with 0.01 µg/ml LPS. TNF-α production induced by dLOS (0.01 µg/ml) was 1.4 times higher than TNF-α production induced by MPL, but did not increase any further up to 1 µg/ml of dLOS. IL-6 and IL-12 secretion in response to dLOS (0.01 µg/ml) were more than 10-fold higher compared with the response to an equal concentration of MPL, but reached a plateau at 1 µg/ml of dLOS. IL-12 is a typical Th1-type cytokine, and IL-6 has a crucial role in functional cytotoxic T lymphocyte (CTL) differentiation. Thus, these results suggest that dLOS is effective at inducing a Th1-type immune response, especially functional CTL activity, while also inducing a limited level of inflammatory response.

**Figure 4:**
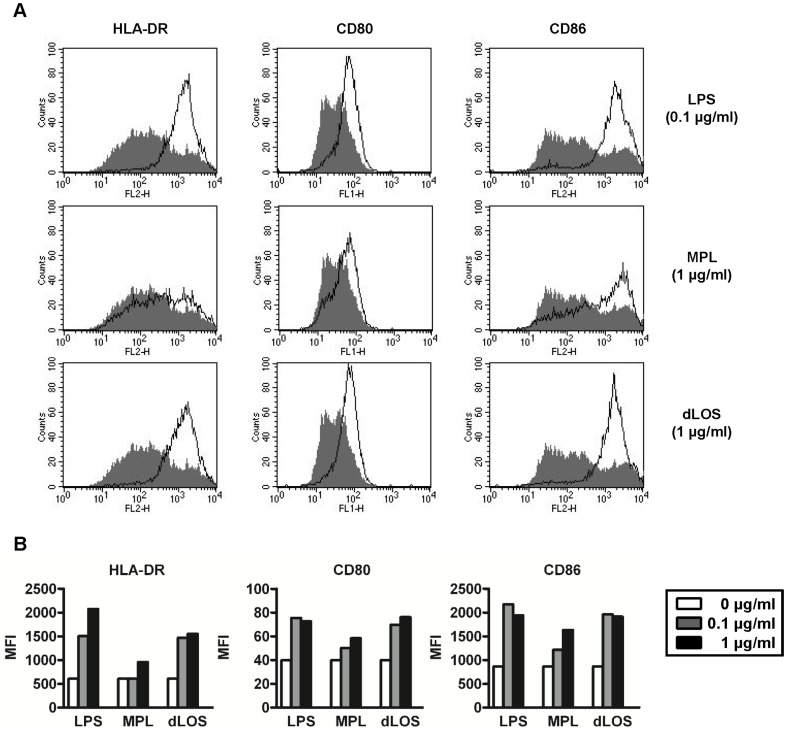
Up-regulation of co-stimulatory molecule expression on human MDDCs. (A) Human MDDCs were stimulated for 24 h with LPS, MPL, or dLOS, and expression of HLA-DR, CD80, or CD86 on CD14-negative populations was assessed using flow cytometry (□). Unstimulated splenocytes (▪). (B) Mean fluorescence intensity (MFI) results. Data represent two independent experiments with similar results.

To determine whether dLOS also affects human MDDC maturation, we stimulated cells with LPS, dLOS, or MPL, and analyzed surface marker expression of HLA-DR, CD80, and CD86 on CD14-negative cells (Figure 4). dLOS up-regulated the expression of co-stimulatory molecule HLA-DR, CD80, and CD86, to a level similar to LPS, but higher than MPL. A higher cytokine IL-12 level secreted from the cells treated with dLOS also indicated a stronger activity for dLOS compared with MPS (data not shown). Altogether, these results demonstrated that dLOS is more active than MPL in inducing maturation of mouse and human DCs.

### Determination of the polarization of T cell responses induced by dLOS

**Figure 5:**
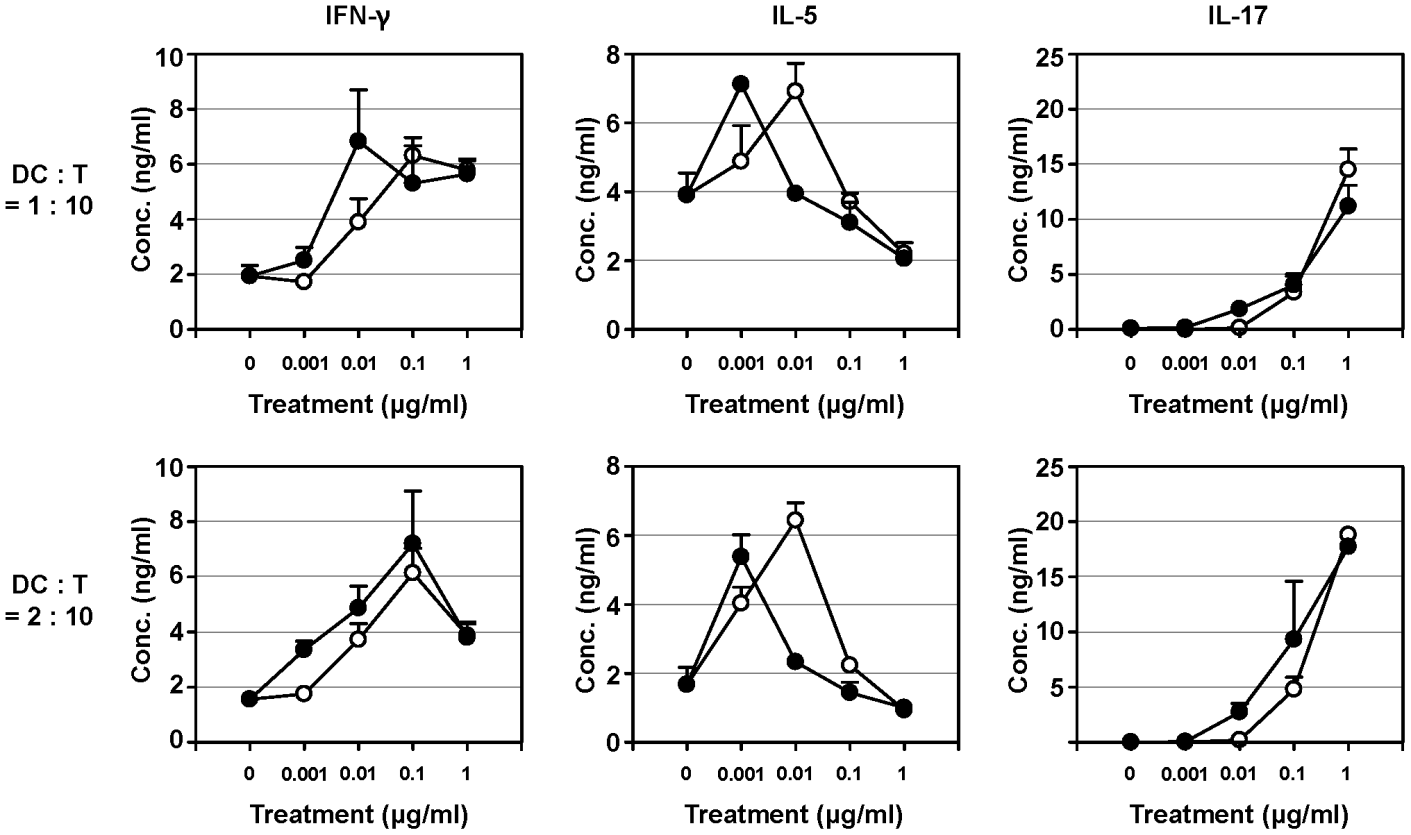
Activation of T cell response to allogeneic DCs stimulated with dLOS. BMDCs from C57BL/6 mice were stimulated with dLOS (•) or MPL (○) at various concentrations for 24 h and co-cultured for 5 days with naïve CD4-positive T cells isolated from BALB/c mice. Culture supernatants were harvested and analyzed for IFN-γ, IL-5, and IL-17 produced by activated T cells. Data are expressed as mean ± SD of values obtained from triplicate reactions and represent two similar experiments.

To determine the polarization of T cell responses induced by dLOS, we examined allogeneic T cell responses to DCs activated by dLOS. Naïve CD4 T cells isolated from BALB/c mice were cocultured with BMDCs (C57BL/6 mice) that were activated with dLOS or MPL. Secreted levels of IFN-γ, IL-5, and IL-17 were assessed (Figure 5). At a low concentration (0.001 µg/ml), dLOS promoted the secretion of IL-5, a typical Th2-type cytokine. However, at a higher concentration IL-5 secretion rapidly declined to, or lower than, the level of untreated cells. The secretion of IFN-γ, a Th1-type cytokine, increased up to 0.01 µg/ml dLOS and then decreased. The secretion of IL-17, a Th17-type cytokine, increased in a dose-dependent manner up to the highest dose (1 µg/ml) of dLOS. The patterns of the three cytokines induced by MPL were similar to the patterns obtained with dLOS except that, for MPL, the peak responses for IFN-γ and IL-5 occurred at a higher concentration than for dLOS. Assessment of IL-12 secreted from C57BL/6 BMDC revealed that there were 10-fold higher levels in dLOS-treated cells, which accounts for the difference in T cell cytokines (Figure S3). These results indicate dLOS induces Th1, Th2, and Th17 responses. The degree of polarization of T cell responses are influenced by the concentration of dLOS.

### TLR4-dependency of dLOS immunostimulatory activity

**Figure 6:**
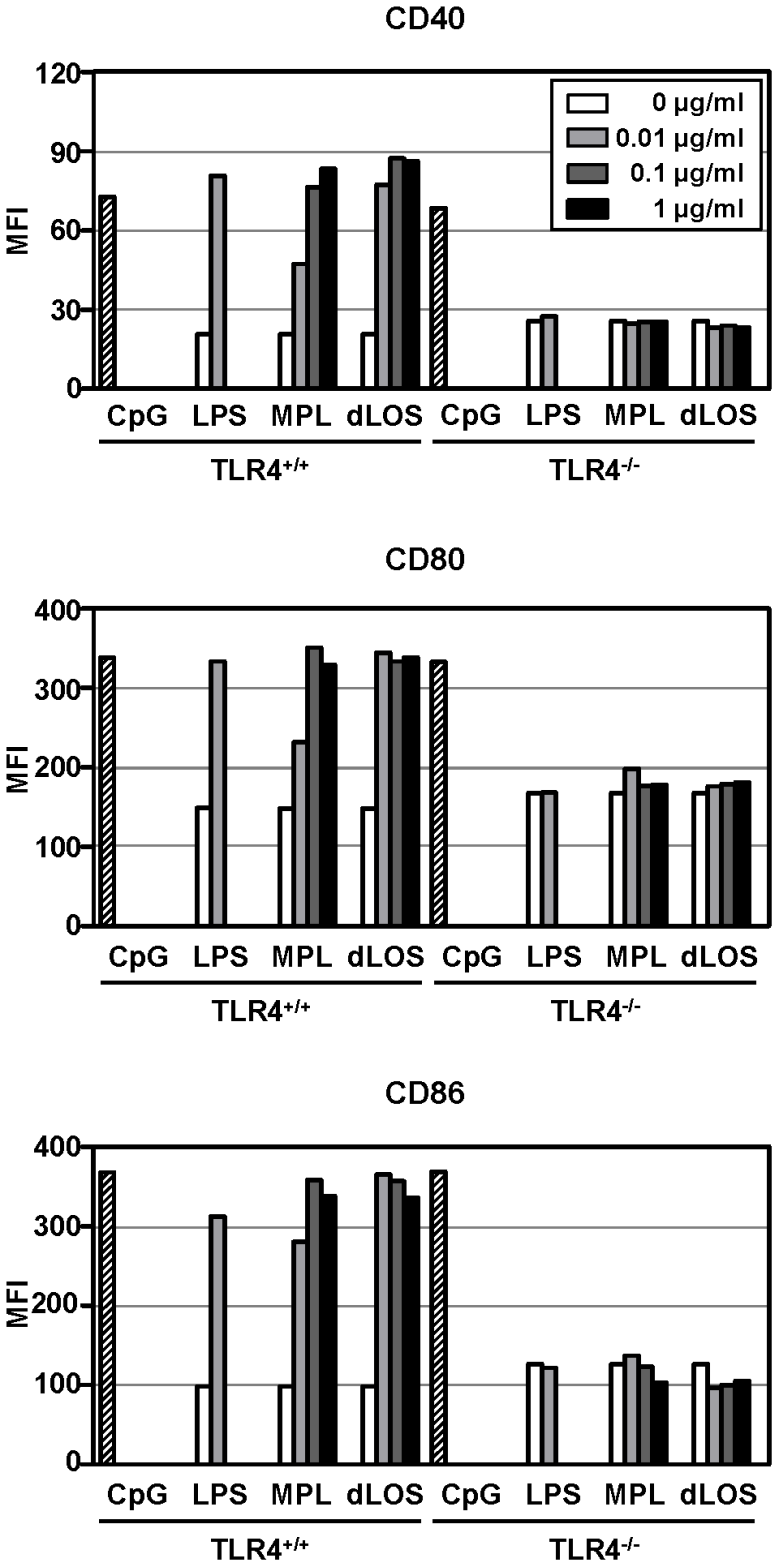
TLR4-dependence of dLOS activity on mouse BMDC maturation. BMDCs isolated from *TLR4^+/+^* and *TLR4^−/−^* BALB/c mice were stimulated for 24 h with LPS, MPL, dLOS, or medium alone. Expression of CD40, CD80, or CD86 on CD11c- positive populations was assessed by flow cytometry. CpG oligonucleotide (10 µg/ml) was used as a positive control.

LPS and lipid A derivatives exert their activity through the TLR4 signaling pathway. dLOS contains only two acyl groups, which may interfere with TLR4-mediated activity. Thus, we decided to determine whether the biological activity of dLOS is TLR4-dependent. BMDCs isolated from TLR4-wild type or *TLR4^−/−^* mice were stimulated with LPS, MPL, or dLOS, and cell surface expression of CD40, CD80, and CD86 on CD11c-positive cells was assessed (Figure 6). Consistent with the data presented in Figure 3, LPS, MPL, and dLOS increased expression of co-stimulatory molecules on BMDCs in the wild type mice. None induced surface molecule expression in the BMDCs from *TLR^−/−^* mice, while CpG, a TLR9 agonist, displayed strong activity in both cell types. Cytokine secretion was also assessed to confirm DC activation. Increased secretion of TNF-α and IL-12 were observed in supernatant of wild type BMDCs stimulated with LPS, MPL, or dLOS, but neither cytokine was detected in the supernatant of TLR4-deficient BMDCs (data not shown). CpG-treated cells induced the secretion of both cytokines regardless of TLR4 expression.

TLR4-dependent dLOS activity was also examined in B cells from TLR4-deficient mice. In the wild type B cells, cell surface expression of the MHC class II molecule and CD86 were increased by exposure to LPS, MPL, or dLOS in a dose-dependent manner (Figure S2). In *TLR4^−/−^* B cells, there was no induction of the MHC class II molecule or CD86 expression. IL-4 used as a positive control increased expression in both cell types. In contrast, LPS had a low activity even in the TLR4-deficient B cells. These results suggest that there is an alternative LPS-activated pathway, presumably a polyclonal activation by LPS O-antigen [24]. TLR4-mediated stimulation by dLOS was also confirmed in mouse peritoneal macrophages (data not shown). Taken together, these data confirmed that dLOS is functionally dependent on TLR4 expression on immune cells.

### *In vivo* induction of cytokine secretion by dLOS

**Figure 7:**
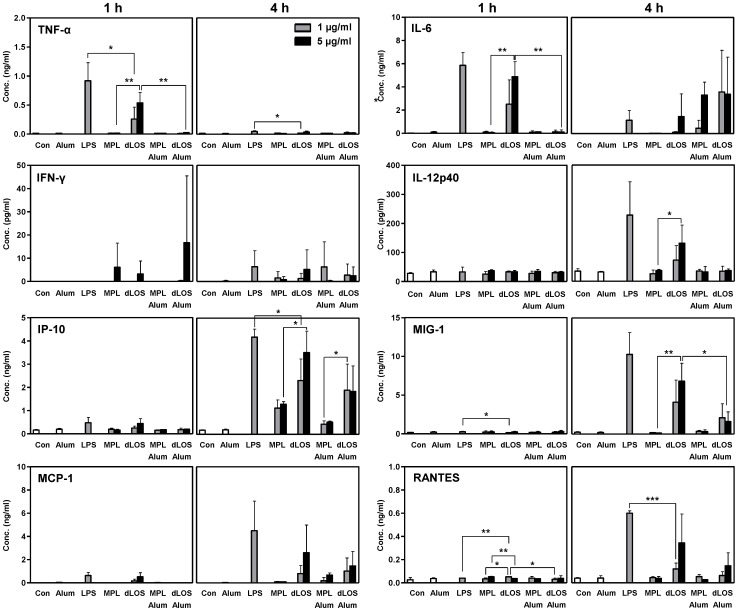
Serum cytokine profiles. BALB/c mice (*n* = 3) were given an intramuscular injection with LPS (1 µg), MPL (1 or 5 µg), or dLOS (1 or 5 µg) alone or in combination with alum at a ratio of 1∶50. Control and alum groups were given saline and 250 µg alum, respectively. Blood was collected 1 h or 4 h after the injection and individual serum samples were assessed for cytokine levels using multiplex cytokine assays. Data are expressed as mean ± SD of values obtained from three mice. Statistical significance between experimental groups; ^*^, *P*<0.05; ^**^, *P*<0.01; ^***^, *P*<0.001. Statistical difference between LPS- and MPL-treated groups is not shown in the figure.

We further investigated dLOS immunostimulatory activity *in vivo*. An intramuscular injection with dLOS induced the secretion of proinflammatory cytokines TNF-α and IL-6, Th1-type cytokines IFN-γ and IL-12, and chemokines IFN-γ-inducible protein 10 (IP-10), gamma interferon-1 (MIG-1), monocyte chemotactic protein-1 (MCP-1), and regulated on activation, normal T cell expressed and secreted (RANTES) (Figure 7). In general, dLOS-induced cytokine levels were lower than the levels induced by LPS, but were significantly higher than MPL-induced levels. In contrast, IFN-γ induction was very low in all treated groups and no significant difference was observed among the groups. Coadministration of alum with dLOS significantly reduced the secretion of TNF-α, MIG-1 and RANTES. IL-6 was an exception, but induction seemed to be delayed in the presence of alum. These data demonstrate that dLOS immunostimulatory activity is more potent than MPL *in vivo*. The combination of dLOS and alum significantly reduced induction of proinflammatory cytokines and chemokines and would be sufficient to stimulate innate and adaptive immune responses necessary for vaccine efficacy while not evoking unnecessarily high inflammatory responses.

### The safety of dLOS

**Figure 8:**
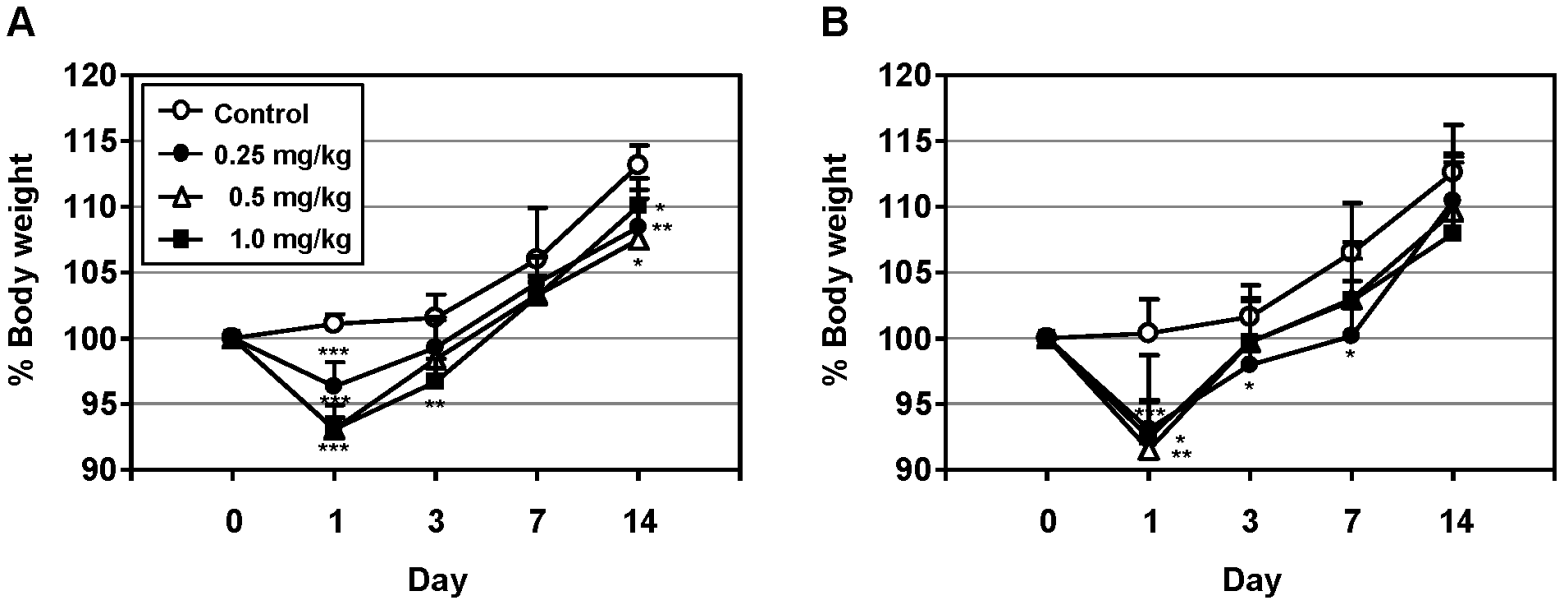
The acute toxicity of dLOS in mice. Groups of five male (A) and five female (B) ICR mice were given a single intramuscular injection with PBS or dLOS at day 0. Body weight change and mortality were monitored for 2 weeks. Data are expressed as mean ± SD of five mice. ^*^, *P*<0.05; ^**^, *P*<0.01; ^***^, *P*<0.001 as compared with PBS control group.

In the dLOS acute toxicity test, no abnormal changes in general appearance or behavior were observed in any of the test animals. For all dLOS doses, body weight was significantly reduced in both male and female mice at day 1 post-injection, compared with the animals given PBS alone (Figure 8). At day 3, however, they began to gain weight and recovered initial body weight by day 7. No gross changes in any of the treated animals were observed at necropsy, suggesting that the reduction was not associated with dLOS toxicity. Based on these data, we concluded that the lethal dose of dLOS is greater than 1 mg/kg body weight.

dLOS is being developed as a human vaccine adjuvant in combination with alum. Therefore, we determined the pyrogenicity of dLOS combined with alum. Three rabbits were administered dLOS plus alum, and individual responses were measured after treatment. The pyrogenicity test results were 0.25°C, 0.10°C, and 0.00°C. The summed response for the three rabbits was 0.35°C, which is below the threshold criteria for a positive pyrogenicity test (1.15°C). This result indicated that dLOS combined with alum is not pyrogenic upon intravenous injection.

## Discussion

LPS is a major component of the outer membrane of Gram-negative bacteria and consists of an amphipathic lipid A portion, a core OS, and an O-antigenic polysaccharide [1]. Of the three, lipid A is most responsible for induction inflammatory responses and septic shock. It exerts its activity via the TLR4-signaling pathway, and thus several different forms of TLR4 agonists with reduced endotoxic activity have been developed as human vaccine adjuvants. All of them, naturally derived or synthetic, are based on the lipid A moiety with modification in the number of phosphate and/or acyl groups [4], [6], [7], [25], [26].

We prepared LOS from an *E. coli* LPS mutant strain and removed fatty acids from lipid A using alkaline hydrolysis of LOS. We used the method described by Bhattacharjee *et al.*, which removes the ester-linked C-12 and C-14 fatty acids via de-*O*-acylation, and leaves only two amide-linked fatty acids [18]. Unlike O-polysaccharide, the core OS region of enterobacteriaceae LPS is relatively conserved. The core OSs of *E. coli* LPS are classified into five categories, K12, R1, R2, R3, and R4 [21], [27]. In this study, we found that the *E. coli* parent strain has the *waaD* gene, which is present only in R3 core type strains [20], [21]. In contrast, the rough mutant strain used to prepare LOS had lost the *waaD* gene. This difference accounted for the truncation of O-side chains of LPS. We also confirmed the presence of the core OS in dLOS using an LPS core-binding mAb, WN1 222-5. WN1 222-5 was developed to treat sepsis caused by enterobacterial infections [22]. The minimum required structure of LPS for high affinity binding to WN1 222-5 is eight sugar residues with four phosphates, i.e., a diphosphorylated heptose trisaccharide, Kdo disaccharide, a nonreducing end glucose, and the phosphorylated glucosamine backbone [28]. It is notable that the LPS-binding site of WN1 222-5 is similar to the LPS inner core-binding site of TLR4 [29]. The lipid A moiety does not contact TLR4 but is mainly recognized through the acyl chains by myeloid differentiating factor 2 (MD-2) in TLR4-MD2 complex [30].

Natural preparations of MPL derived from *S. minnesota* R595 consist of a mixture of molecules containing three to seven acyl groups [31]. We also observed several peaks with ∼200 Da lower mass after a major peak on the mass spectrum for MPL, which represented heterogeneous molecular species with various numbers of acyl groups (data not shown). There was only one predominant peak at 2826.67 *m/z* for dLOS on the mass spectrum, which suggests that the dLOS preparation is relatively homogenous and consists of a major molecular species of molecular mass 2826.67 Da. These data led us to conclude that dLOS consists of nine sugar residues of core OS and a lipid A backbone with two phosphates and two *N*-linked, but not *O*-linked, fatty acids.

Because of the immunostimulatory activity (especially on dendritic cells) that plays an essential role linking innate immunity to adaptive immunity, LPS-derivatives have been developed as vaccine adjuvants. All of them are naturally derived or synthetic lipid A derivatives. Studies on the relationship between lipid A structure and its activity and function revealed that lipid A analogs vary greatly in their biological activity. The shape, charge and physical structure of lipid A affect biological activity; only conical molecules, but not cylindrical ones, are biologically active [32], [33]. The number of phosphate groups, and the symmetry, number, and length of fatty acyl chains are also critical for immune stimulating activity [8], [23]. Dephosphorylation of lipid A at the 1-position contributes to the low activity of MPL. Acyl chain removal also greatly reduces activity; penta-acyl lipid A has a 10–100 fold decreased activity. Synthetic tetra-acyl lipid A (lipid IVa) and tri-acyl lipid A have no biological activity and instead act as antagonists [9], [23]. In contrast, a naturally derived triacyl lipid A (OM-174) has been shown to retain the ability to induce NO production in murine macrophages [34].

dLOS contains only two *N*-linked acyl groups. We confirmed that it exerts its immunostimulatory activity on various mouse and human immune cells through TLR4-mediated signaling. In the Limulus Amebocyte Lysate assay, the endotoxic activity of dLOS was comparable to MPL that contained six acyl groups with monophosphate at the 4′-position. Unlike lipid A derivatives, dLOS contains the core OS moiety, which implies that the core OS confers biological activity even in the absence of *O*-linked acyl groups. *E. coli* Kdo_2_-lipid A has immunostimulatory activity comparable to LPS; it has the ability to induce cytokines in RAW264.7 cells and BM-derived macrophages [10]. Removal of two Kdo molecules of meningococcal LOS resulted in a 10-fold reduction in the ability to stimulate the human macrophage CD14/TLR4 pathway [11]. Muroi *et al.* reported that a loss of two Kdo molecules on *Salmonella* lipid A but not *E. coli* lipid A drastically reduced nuclear factor-kappa B (NF-κB) activation through human CD14/TLR4/MD-2 [35]. These observations indicate that Kdo plays an important role in initiating TLR4 signaling.

During bacterial infection, LPS extracted from the bacterial membrane is transferred to TLR4 by two accessory proteins, LPS-binding protein and CD14 [36]. It is subsequently transferred to the TLR4 and MD-2 complex, and the LPS binding initiates the dimerization of two TLR4-MD-2 complexes. Studies on the crystal structure of TLR4-MD-2 complexed with LPS or lipid IVa revealed that five of the six lipid chains of LPS are buried deep inside a large hydrophobic pocket in MD-2 [29], [37]. The remaining *N*-linked acyl chain at the 2-position is exposed to the surface of MD-2 and forms a hydrophobic interaction with the conserved phenylalanines of TLR4, which results in dimerization of the complexes. The two phosphate groups on lipid A bind the TLR4-MD-2 complex by interacting with positively charged residues on TLR4 molecules and on MD-2, which suggests that they have an important role in dimerization of the complexes [29]. The LPS inner core forms several bonds with MD-2 and TLR4 molecules, but the significance of these interactions for TLR4 dimerization has not been clearly demonstrated. Considering these observations, two *N*-linked acyl groups on dLOS may not be able to form hydrophobic interactions with TLR4-MD-2 strong enough to efficiently trigger dimerization. However, the core OS of dLOS has rich negative charges from two Kdos and phosphate groups on heptose residues and may contribute to the induction of dimerization of the TLR4-MD-2 complexes and activate TLR4 signaling [22], [29].

LPS-induced TLR4 signaling is bifurcated into two pathways [38]. LPS activation of the myeloid differentiation primary response gene 88 (MyD88)-dependent pathway leads to rapid activation of NF-κB, which in turn controls expression of early inflammatory cytokines such as TNF-α and IL-6 [39]. The activation of the Toll IL-1 receptor domain-containing adaptor-inducing IFN-β (TRIF)-dependent pathway leads to rapid activation of interferon release factor 3 (IRF3), which induces production of type I IFNs and chemokines, including RANTES and IP-10 [40], [41]. Zughaier *et al.* reported that in human and mouse macrophage-like cell lines, *E. coli* LPS selectively induces the MyD88-dependent signaling pathway while *S. minnesota* LPS activates the MyD88-independent pathway [42]. Using an adoptive transfer method, Mata-Haro *et al.* showed that the immunostimulatory activity of MPL is mainly driven by the TRIF-pathway [43]. The same group also reported that activation of the TRIF-pathway is critical for T cell proliferation and survival induced by MPL. Defective type I IFN production results in the loss of its adjuvant effects [44]. The TRIF-pathway is important for CD86 and CD40 upregulation on splenic DCs, but both TRIF and MyD88 pathways are required for CD80 upregulation [44]. On the other hand, MacLeod *et al.*, reported that MPL induces production of a MyD88 cytokine IL-6, which is critical for effective cytotoxic T cell differentiation [45]. Furthermore, dual signaling of MyD88 and TRIF is required for maximal DC maturation, and MyD88 has a larger role than TRIF for maximal T cell priming by LPS-matured DCs [46]. Studies on a synthetic TLR4 agonist, GLA-SE adjuvant, demonstrated that a synergistic interaction between MyD88 and TRIF signaling leads to Th1-polarization of the immune response induced by the adjuvant [47]. In the present study, we observed that dLOS enhanced the expression of the surface co-stimulatory molecules CD40, CD86, and CD80 on mouse BMDCs. dLOS also induced the production of MyD88- and TRIF-dependent cytokines. These results indicate that dLOS-induced TLR4 activation triggers MyD88- and TRIF-dependent signaling pathways, though much lower in activity when compared with LPS.

In addition to activity, adjuvant safety is a major concern. In allogeneic T cell response assays, we observed that dLOS induced IL-17 secretion in a dose-dependent manner. IL-17 is believed to play a role in autoimmune diseases, while it is required for protection against pathogenic infections, especially *Mycobacterium tuberculosis* infection [48]. The IL-17 level induced by dLOS was similar at all concentrations tested to that of MPL, which is currently used in human vaccines but has not been implicated in autoimmune diseases up to the present. By analogy, dLOS is not likely to cause autoimmunity in humans. We also assessed dLOS safety in various systems. The results of the acute toxicity study in mice indicated that dLOS was not toxic up to 1.0 mg/kg body weight. Toxicity studies in rats and beagles have confirmed the safety of dLOS alone or dLOS plus alum (data not shown). A combination of dLOS and alum, designated as CIA06, was formulated with human papillomavirus L1 VLPs to develop an HPV vaccine that is currently in a phase I clinical trial.

In summary, our results indicate that dLOS consists of a core OS lacking the terminal glucose residue, and a glucosamine disaccharide containing two phosphates and two *N*-linked acyl groups. The results also suggest that, in the presence of the core OS, *O*-linked acyl groups of LPS are dispensable for activating the TLR4 signaling pathway. In addition, dLOS stimulates both MyD88- and TRIF-dependent pathways downstream of the TLR4 signaling. The data obtained in this study also provide the basis for further development of dLOS as a safe and effective adjuvant for human vaccines.

## Supporting Information

Figure S1 **dLOS stimulation of mouse B cell proliferation.** Splenocytes from BALB/c mice were stained with CFSE, incubated with LPS, MPL, or dLOS for 3 days, and stained with anti-B220-PerCP mAb followed by flow cytometry. Histograms are derived from the B220-positive cells. Unstimulated splenocytes (▪).
(TIF)

Figure S2 **Surface marker expression of TLR4-mediated B cell activation by dLOS.** Splenocytes from *TLR4^+/+^* and *TLR4^−/−^* mice were cultured in the presence of LPS, MPL, dLOS, or media alone, for 2 days. Cells were harvested and examined for expression of MHC class II and CD86 molecules on B220-positive cell population using flow cytometry. Unstimulated splenocytes (▪). Data represent three independent experiments with similar results. Mouse IL-4 (500 U/ml) was included as a positive control. Data represent three independent experiments with similar results.
(TIF)

Figure S3 **IL-12 secretion from BMDCs from C57BL/6 mice treated with dLOS and MPL.** BMDCs were isolated from C57BL/6 mice, stimulated with dLOS (•) or MPL (○) at various concentrations for 24 h, and secreted IL-12 levels were assessed using sandwich ELISA.
(TIF)

Methods S1 (DOC)
