# Correction: Characterization of the Structure and Immunostimulatory Activity of a Vaccine Adjuvant, De-*O*-Acylated Lipooligosaccharide

**DOI:** 10.1371/journal.pone.0094517

**Published:** 2014-04-01

**Authors:** The PLOS ONE Staff

The authors would like to provide a clarification in relation to Figure 1D:

**Figure 1:**
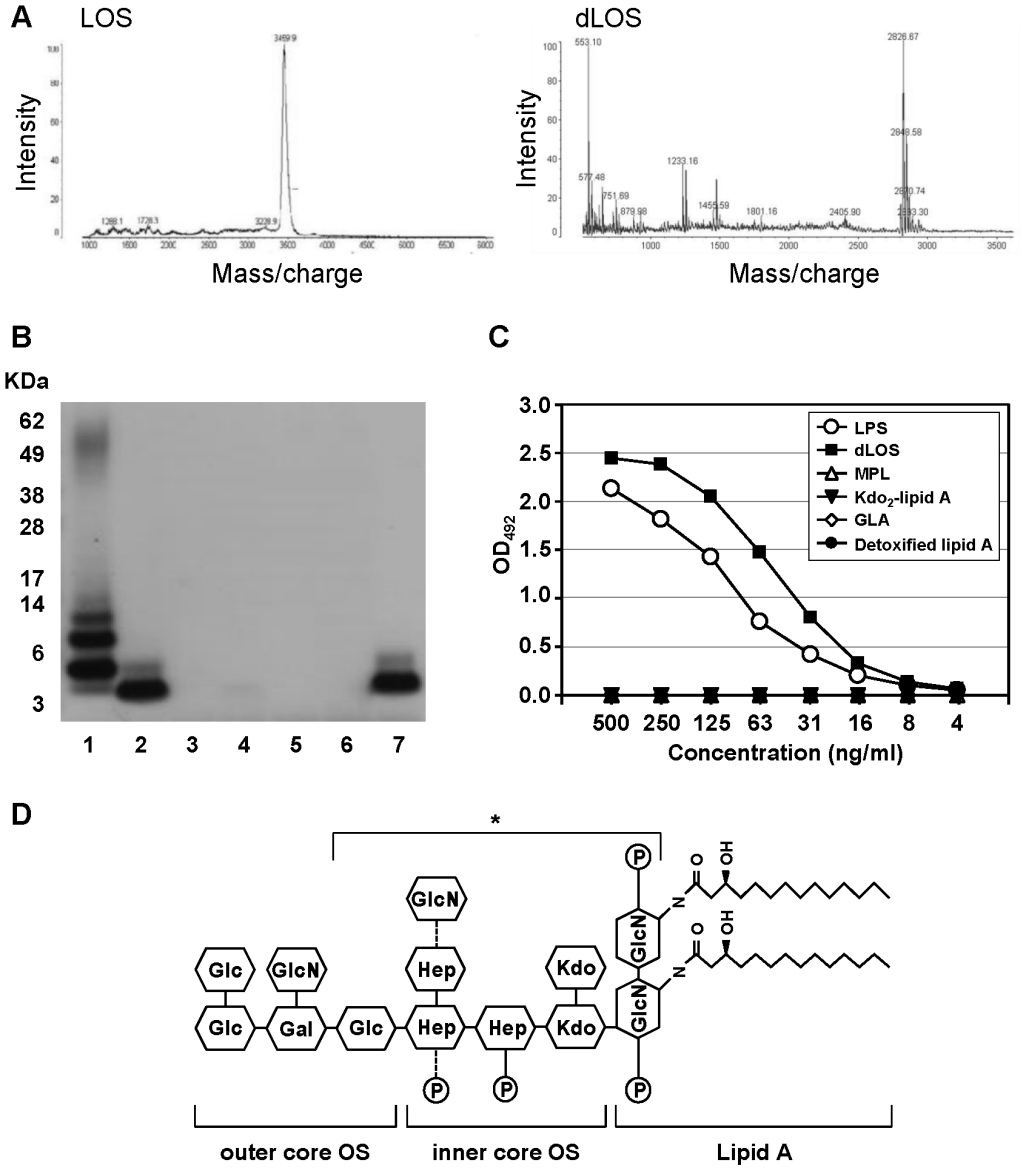
Characterization of dLOS structure. (A) Mass spectra for *E. coli* LOS and dLOS. (B) and (C) Reactivity of LPS, dLOS, and four lipid A derivatives with an mAb specific for the core OS region of LPS as determined by immunoblot analysis and ELISA, respectively. Lanes on the immunoblot: 1, LPS 2.5 μg; 2, dLOS 1 μg; 3, MPL 4 μg; 4, Kdo2-lipid A 4 μg; 5, GLA 4 μg; 6, detoxified lipid A from S. minnesota R595 4 μg; 7, dLOS 1 μg. The positions of protein size markers are marked on the left side of the gel. Data in B and C represent two and four independent experiments, respectively, with similar results. (D) The predicted chemical structure of dLOS. In the inner core OS, either glucosamine or phosphate is linked to the third heptose or the second heptose (marked with dotted lines), respectively. The WN1 222-5 antibody binding site is denoted by an asterisk.

“In Figure 1D, we characterized dLOS which we developed as a vaccine adjuvant. To determine the structure of dLOS, the whole genome of the *E. coli* LPS mutant strain was sequenced by a CRO, who made mistakes while analyzing the gene data. According to the original data, the mutant strain had a mutation in the waaD gene, which would remove the terminal glucose residue of the outer core oligosaccharide. However, it was confirmed that the strain contained the gene intact. Therefore, we believe that dLOS contains the whole core OS structure. We corrected the predicted structure of dLOS in Figure 1D and also revised the text and figure legend accordingly.”

The sixth sentence of the Abstract should read: “dLOS consists of the R3-type core, a glucosamine disaccharide with two phosphate groups, and two N-linked acyl groups.”

The second paragraph of the Characterization of dLOS chemical structure subsection of the Results section should read: “To investigate the dLOS carbohydrate core structure, we performed whole genome sequencing of the *E. coli* LPS mutant strain. The results revealed that the strain contained the *waaD*, *waaI* and *waaJ* genes, which are present only in R3 type core strains among *E. coli* strains. This result suggested that dLOS contains the R3 core-oligosaccharide [20, 21]. We confirmed the presence of the core OS moiety by immunoblot analysis using mAb WN1 222-5, which is specific for the LPS core region [22]. WN1 222-5 bound to LPS and dLOS but not to four lipid A derivatives (Figure 1B). Gel results also clearly showed that dLOS is smaller in size than the lipid A-core OS of LPS. We used ELISA to compare the reactivity of WN1 222-5 with LPS, dLOS, and lipid A derivatives, and found that the antibody was highly reactive with LPS and dLOS but not with other lipid A derivatives (Figure 1C). A higher optical density of dLOS may be attributed to a greater molar ratio of dLOS compared with LPS. These results confirmed that dLOS retained the core OS moiety. Based on these data, we predicted the chemical structure of dLOS (Figure 1D). It contains the R3-type core OS that includes a heptose-phosphate epitope and two Kdo residues, and the lipid A containing only two N-linked acyl groups and two phosphates. The endotoxic activity of dLOS determined using the Endosafe®-Portable Test System was 5.4 × 103 EU/mg, which was comparable to that of MPL.”

The fourth sentence of the second paragraph of the Discussion section should read: “In this study, we found that the *E. coli* LPS mutant strain has three genes which are present only in R3 core type strains, confirming that dLOS contains the R3 core OS [20, 21].”

The fifth sentence of the second paragraph of the Discussion section should be removed.

The fourth sentence of the third paragraph of the Discussion section should read: “These data led us to conclude that dLOS consists of the core OS and a lipid A backbone with two phosphates and two N-linked, but not O-linked, fatty acids.”

The first sentence of the final paragraph of the Discussion section should read: “In summary, our results indicate that dLOS consists of the R3-type core OS, and a glucosamine disaccharide containing two phosphates and two N-linked acyl groups.”

Please see the corrected legend for Figure 1 here.
