# Supplementary figures and images for: Characterization of the Structure and Immunostimulatory Activity of a Vaccine Adjuvant, De-O-Acylated Lipooligosaccharide
Source: PLoS One. 2014 Jan 22;9(1):e85838. doi: 10.1371/journal.pone.0085838 (PMC3899070; doi:10.1371/journal.pone.0085838)

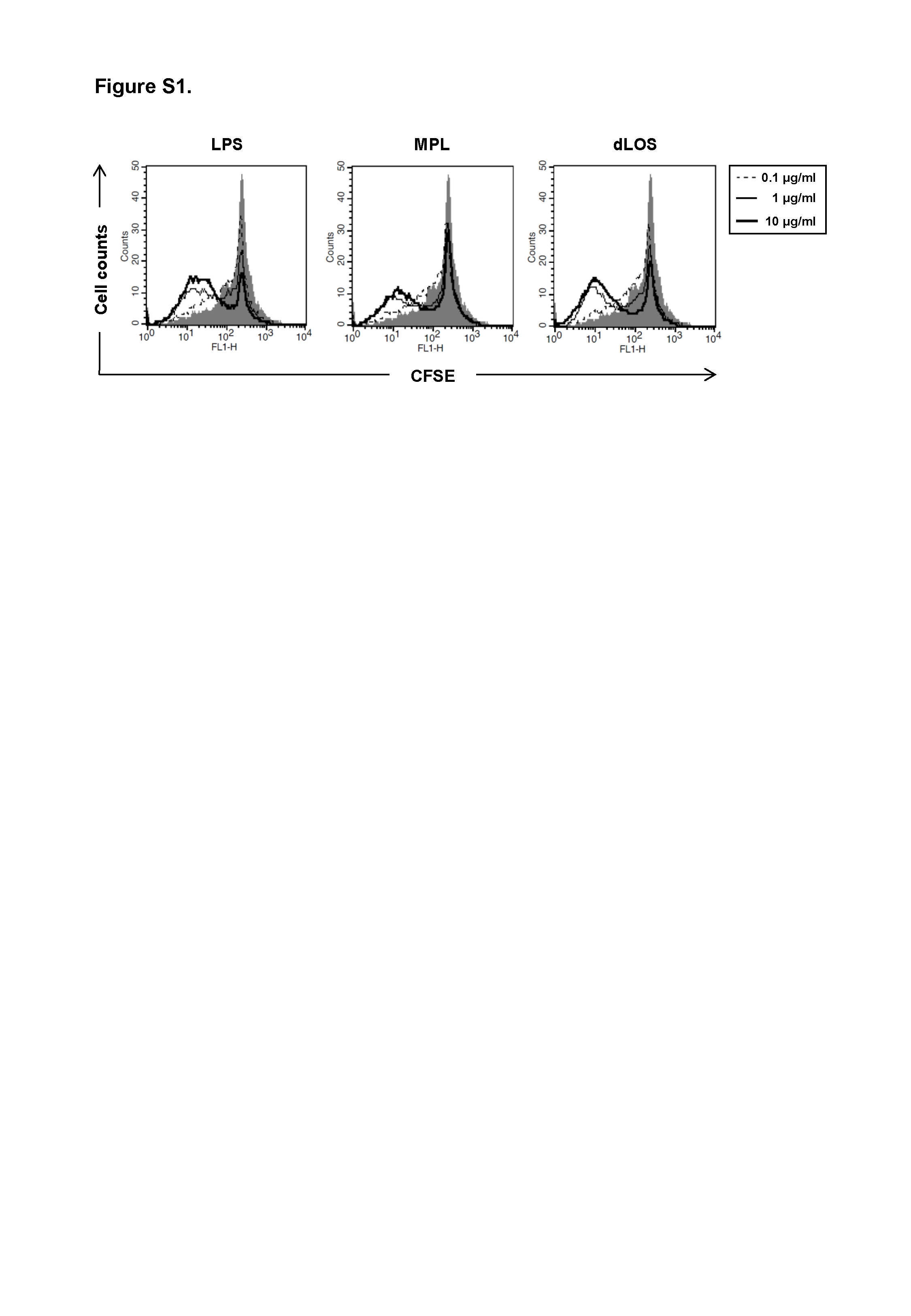

Supplement: Figure S1 — dLOS stimulation of mouse B cell proliferation. Splenocytes from BALB/c mice were stained with CFSE, incubated with LPS, MPL, or dLOS for 3 days, and stained with anti-B220-PerCP mAb followed by flow cytometry. Histograms are derived from the B220-positive cells. Unstimulated splenocytes (▪). (TIF) [file pone.0085838.s001.tif]

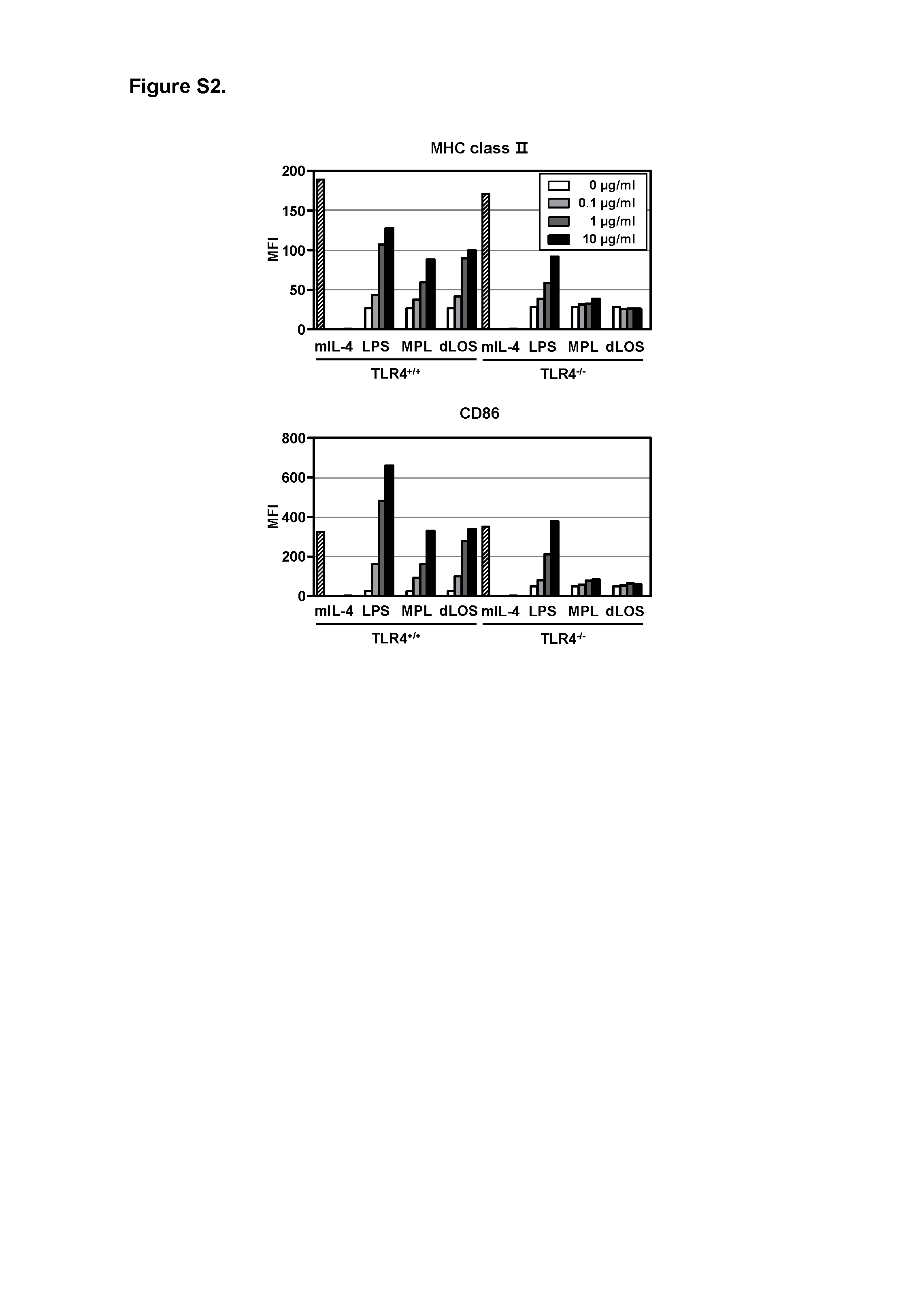

Supplement: Figure S2 — Surface marker expression of TLR4-mediated B cell activation by dLOS. Splenocytes from TLR4+/+ and TLR4−/− mice were cultured in the presence of LPS, MPL, dLOS, or media alone, for 2 days. Cells were harvested and examined for expression of MHC class II and CD86 molecules on B220-positive cell population using flow cytometry. Unstimulated splenocytes (▪). Data represent three independent experiments with similar results. Mouse IL-4 (500 U/ml) was included as a positive control. Data represent three independent experiments with similar results. (TIF) [file pone.0085838.s002.tif]

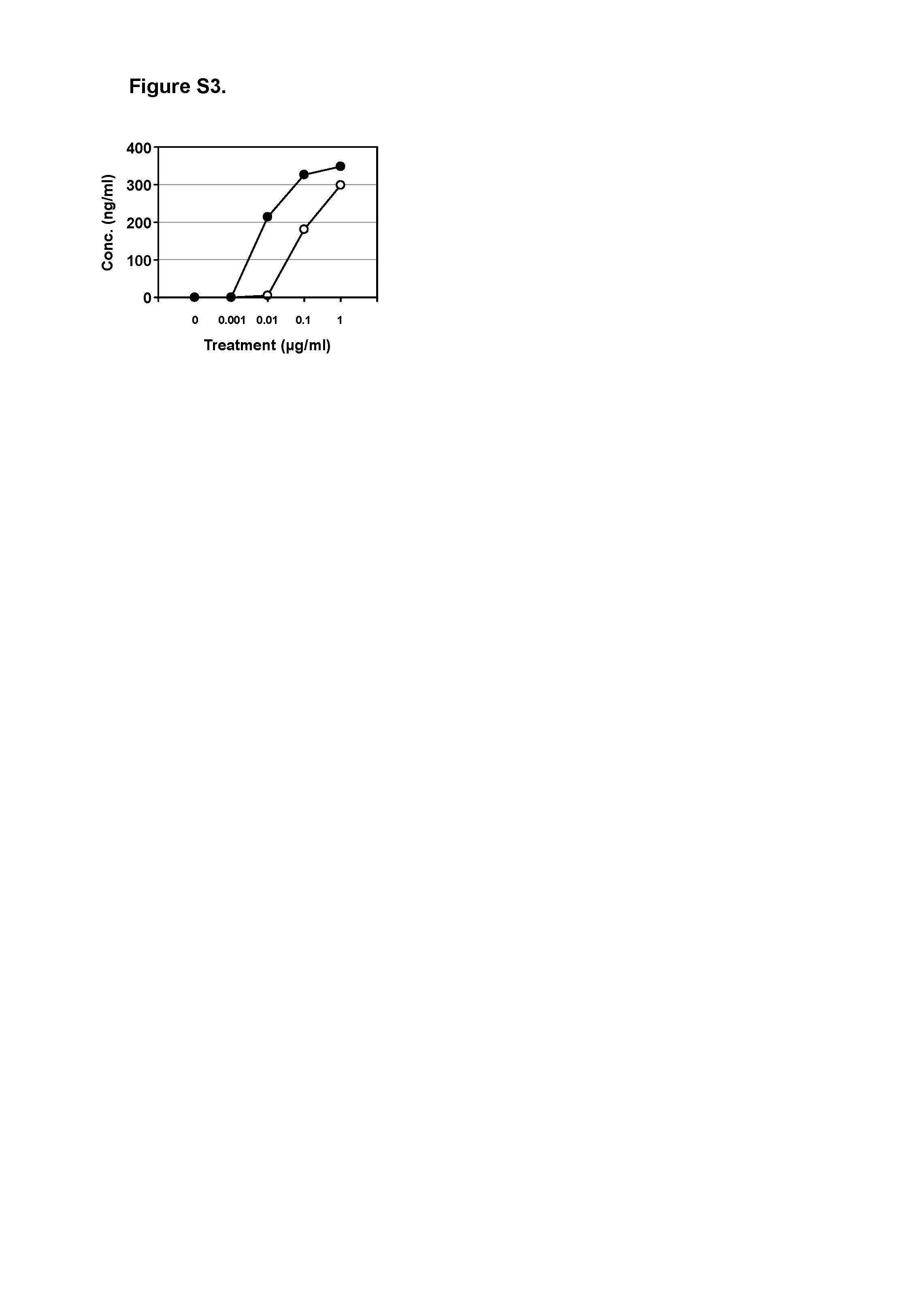

Supplement: Figure S3 — IL-12 secretion from BMDCs from C57BL/6 mice treated with dLOS and MPL. BMDCs were isolated from C57BL/6 mice, stimulated with dLOS (•) or MPL (○) at various concentrations for 24 h, and secreted IL-12 levels were assessed using sandwich ELISA. (TIF) [file pone.0085838.s003.tif]
