# Supplementary material for: Characterization of the Structure and Immunostimulatory Activity of a Vaccine Adjuvant, De-O-Acylated Lipooligosaccharide
Source: PLoS One. 2014 Jan 22;9(1):e85838. doi: 10.1371/journal.pone.0085838 (PMC3899070; doi:10.1371/journal.pone.0085838)
Supplement: Methods S1 — (DOC) [file pone.0085838.s004.doc]

**Methods for supplementary data**

**Measurement of B cell proliferation**

To determine B cell proliferation, mouse splenocytes were stained with CFSE (5 μM) and cultured in the presence of LPS, MPL, or dLOS for 3 days. The cells were harvested and stained with anti-B220-PerCP mAb, followed by flow cytometry. B220-positive cells were gated, and the intensity of CFSE was analyzed.

**Measurement of B cell surface molecule expression in *TLR4^+/+^* and *TLR4^-/-^*mice**

Mouse splenocytes were isolated from *TLR4^+/+^* and *TLR4^-/-^*BALB/c mice. After RBC lysis, splenocytes were seeded at a density of 1 × 10^6^ cells/ml and cultured for 48 h in the presence of LPS, MPL, or dLOS. Positive control cells were treated with mouse IL-4 (500 U/ml). The cells were harvested and stained with anti-B220-PerCP mAb plus anti-I-A-FITC mAb or anti-CD86-PE mAb, followed by flow cytometric analysis. B220-positive cells were gated, and expression of MHC class II and CD86 was analyzed.

**Supporting information legends**

**Figure S1. dLOS stimulation of mouse B cell proliferation.** Splenocytes from BALB/c mice were stained with CFSE, incubated with LPS, MPL, or dLOS for 3 days, and stained with anti-B220-PerCP mAb followed by flow cytometry. Histograms are derived from the B220-positive cells. Unstimulated splenocytes (■).

**Figure S2. Surface marker expression of TLR4-mediated B cell activation by dLOS.** Splenocytes from *TLR4^+/+^* and *TLR4^-/-^* mice were cultured in the presence of LPS, MPL, dLOS, or media alone, for 2 days. Cells were harvested and examined for expression of MHC class II and CD86 molecules on B220-positive cell population using flow cytometry. Unstimulated splenocytes (■). Data represent three independent experiments with similar results. Mouse IL-4 (500 U/ml) was included as a positive control. Data represent three independent experiments with similar results.

**Figure S3. IL-12 secretion from BMDCs from C57BL/6 mice treated with dLOS and MPL.** BMDCs were isolated from C57BL/6 mice, stimulated with dLOS (●) or MPL (○) at various concentrations for 24 h, and secreted IL-12 levels were assessed using sandwich ELISA.
